# Role of elevated red cell distribution width on acute kidney injury patients after cardiac surgery

**DOI:** 10.1186/s12872-018-0903-4

**Published:** 2018-08-14

**Authors:** Zhouping Zou, Yamin Zhuang, Lan Liu, Bo Shen, Jiarui Xu, Wuhua Jiang, Zhe Luo, Jie Teng, Chunsheng Wang, Xiaoqiang Ding

**Affiliations:** 1 Department of Nephrology, Zhongshan Hospital, Shanghai Medical College, Fudan University, No 180 Fenglin Road, Shanghai, 200032 China; 2 Shanghai Medical Center of Kidney, No 180 Fenglin Road, Shanghai, 200032 China; 3 Shanghai Institute for Kidney and Dialysis, No 180 Fenglin Road, Shanghai, 200032 China; 4 Shanghai Key Laboratory of Kidney and Blood Purification, No 180 Fenglin Road, Shanghai, 200032 China; 5 Department of Critical Care Medicine, Zhongshan Hospital, Shanghai Medical College, Fudan University, No 180 Fenglin Road, Shanghai, 200032 China; 6 Hemodialysis Quality of Control Center of Shanghai, No 180 Fenglin Road, Shanghai, 200032 China; 7 Department of Nephrology, Xiamen Branch, Zhongshan Hospital, Fudan University, No 668 Jinhu Road, Xiamen, 361015 Fujian China; 8 Department of Cardiovascular Surgery, Zhongshan Hospital, Shanghai Medical College, Fudan University, No 180 Fenglin Road, Shanghai, 200032 China

**Keywords:** Acute kidney injury, Cardiac surgery, Prognosis, Red cell distribution width

## Abstract

**Background:**

The aim of the study was to explore associations between elevated red cell distribution width (RDW) and acute kidney injury (AKI) in patients undergoing cardiac surgery (CS-AKI).

**Methods:**

Preoperative, intraoperative and postoperative data of 10,274 patients undergoing cardiac surgery, including demographic data, were prospectively collected from January 2009 to December 2014. Propensity score matching was used on the basis of clinical characteristics and preoperative variables. An elevated RDW was defined as the difference between RDW 24 h after cardiac surgery and the latest RDW before cardiac surgery.

**Results:**

A total of 10,274 patients were included in the unmatched cohort, and 3146 patients in the propensity-matched cohort. In the unmatched cohort, the overall CS-AKI incidence was 32.8% (*n* = 3365) with a hospital mortality of 5.5% (*n* = 185). In the propensity-matched cohort, the elevated RDW in AKI patients was higher than in patients without AKI (0.3% (0.0%, 0.7%) vs 0.5% (0.1, 1.1%), *P* <  0.001) and the elevated RDW incidences were 0.4% (0.1%, 0.9%), 0.6% (0.2%, 1.1%) and 1.1% (0.3%, 2.1%) in stage 1, 2 and 3 AKI patients (*P* <  0.001). Among propensity-matched patients with CS-AKI, the level of elevated RDW in non-survivors was higher than in survivors [1.2% (0.5%, 2.3%) vs 0.5% (0.1%, 1.0%), *P* <  0.001] and a 0.1% increase in elevated RDW was associated with a 0.24% higher risk of within-hospital mortality in patients with CS-AKI. Estimating the receiver-operating characteristic (ROC) area under the curve (AUC) showed that an elevated RDW had moderate discriminative power for AKI development (AUC = 0.605, 95% CI, 0.586–0.625; *P* <  0.001) and hospital mortality (AUC = 0.716, 95% CI, 0.640–0.764; *P* <  0.001) in the propensity-matched cohort.

**Conclusions:**

An elevated RDW might be an independent prognostic factor for the severity and poor prognosis of CS-AKI.

## Background

Red blood cell distribution width (RDW), which is a marker that describes the morphology of red blood cells and is routinely reported in complete blood counts, is a measurement of erythrocyte variability and heterogeneity. RDW can be expressed either in absolute values (RDW-SD) or as a percentage (RDW %); the latter approach is more widely used in routine laboratory practice. RDW s together with the value of mean corpuscular volume (MCV) to classify, diagnose and differentiate the causes of anemia, especially iron-deficiency anemia [1]. Recently, several studies have shown that a high RDW level is a strong independent predictor of increased morbidity and mortality in patients with heart failure, myocardial infarction, paroxysmal atrial fibrillation, primary biliary cirrhosis, chronic hepatitis C, pulmonary embolism, chronic obstructive pulmonary disease (COPD), leukoaraiosis and drug-eluting stent restenosis [2–9]. However, there is limited research regarding the potential association between elevated RDW and the development, severity and prognosis of AKI associated with cardiac surgery (CS-AKI). The present study investigated the potential association between elevated RDW and the development and prognosis of CS-AKI patients in order to identify a novel biomarker for the early diagnosis, clinical severity and prognosis of CS-AKI.

## Methods

### Study population

We retrospectively analyzed data from patients who underwent cardiac surgery from January 2009 to December 2014 in the Zhongshan Hospital affiliated to Fudan University and matched them 1:1 based on propensity scoring. The criteria for exclusion were: age (< 18 years), anemia (hemoglobin < 120 g/L for males; hemoglobin < 110 g/L for females), solitary kidney or history of kidney transplants, severe renal dysfunction with estimated glomerular filtration rate (eGFR) ≤ 30 mL/min/1.73 m^2^ at baseline; preoperative circulatory assist devices, preoperative renal replacement therapy (RRT) or those undergoing a heart transplant; insufficient examination results at baseline.

### AKI and RDW definitions

AKI was defined according to the Kidney Disease Improving Global Outcomes (KDIGO) [10] criteria as described recently [11]: 1) Increase in serum creatinine (Scr) by ≥0.3 mg/dL (≥ 26.5 μmol/L) within 48 h; 2) Increase in Scr to ≥1.5 times baseline, which is known or presumed to have occurred within the prior 7 days; 3) Urine volume <  0.5 mL/kg/h for 6 h and staged according to the Scr and urine output. An elevated RDW was defined as the difference between the RDW value 24 h after cardiac surgery and the latest RDW value before cardiac surgery. The reference range for RDW value was 11.0–16.0% at the Zhongshan Hospital affiliated to Fudan University.

### Clinical trial design and methods

Informed consent was provided by all of the patients according to hospital guidelines. At admission, baseline blood testing, echocardiography and electrocardiography were performed. Recorded clinical data were preoperative baseline characteristics and blood tests, complications, preoperative renal function (determined by the latest serum creatinine value before cardiac surgery) as well as perioperative data about cardiac functions according to the 1994 New York Heart Association (NYHA) classification and intraoperative variables which included cardiac output data derived from echocardiography, cardiopulmonary bypass (CPB) and aortic cross-clamp (ACC) durations in minutes, as well as types of surgery. The recorded postoperative variables were mechanical ventilation duration and urine output during the first post-surgery 24 h as well as AKI incidence and stage in addition to hospital mortality.

### Outcomes

The primary endpoint was the occurrence of AKI. The secondary endpoint was all cause of mortality.

### Statistical analysis

All statistical analyses were performed using SPSS Statistics for Windows (Version 22.0. Armonk, NY: IBM Corp). Propensity score matching was used to adjust for observed differences in characteristics of patients with and without AKI. Comparisons of the differences in the baseline characteristics were performed using a Student’s *t*-test for parametric data and a Mann-Whitney U-test for non-parametric data. Categorical variables were compared using a chi-square test or Fisher’s exact test.

Associations of pre-, intra-, post-operative and demographic data were tested with Wilcoxon rank-sum or two-sample *t*-tests for continuous and a Pearson χ2 test for categorical variables. Continuous variables are presented as mean ± standard deviation or median (interquartile range [IQR]) and categorical variables are shown as frequency counts (%). A multivariable logistic regression model was used for estimation of unadjusted and adjusted odds ratios (ORs). Pearson’s and Spearman correlation tests were used to determine the correlation between variables. Receiver operator characteristic (ROC) curves were constructed to analyze the discriminating power of elevated RDW for predicting the development of AKI and all-cause hospital mortality. ROC curve analysis statistics were determined with area under the curve (AUC) (95% CI) estimated by bootstrapping. Two-tailed *P*-values < 0.05 were considered to be statistically significant for all analyses.

## Results

### Patient characteristics of the study population

#### Unmatched cohort

A total of 10,274 patients undergoing cardiac surgery were enrolled; most were men (56.7%). The overall CS-AKI incidence was 32.8% (*n* = 3365) with a hospital mortality of 5.5% (*n* = 185). The mean age at hospital admission was 53.3 years (± SD 13.9). The mean age, proportion of males mean body mass index (BMI), hemoglobin, white blood cells (WBC), serum creatinine (SCr), blood urea nitrogen (BUN), serum uric acid (SUA), CPB time, elevated RDW, preoperative proportions of hypertension, diabetes mellitus (DM), coronary angiography, chronic heart failure (NYHA > II), aortic cross-clamp (ACC) time were increased in the AKI group, whereas baseline albumin was decreased (Table 1).

**Table 1:** Comparison of the Demographic and Baseline Characteristics of unmatched and matched AKI and non-AKI groups

	Unmatched Cohort (*n* = 10,274)		*P*-value	Matched Cohort (*n* = 3146)		*P*-value
	Non-AKI group (*n* = 6909)	AKI group(*n* = 3364)		Non-AKI group (*n* = 1573)	AKI group (*n* = 1573)	
Age (years)	51.7 ± 14.0	56.8 ± 12.3	< 0.001	54.6 ± 12.0	54.4 ± 12.1	0.782
Male, sex	3584 (51.9%)	2233 (66.6%)	< 0.001	966 (61.4%)	913 (58.0%)	0.059
Comorbidities						
Hypertension	1748 (25.3%)	1177 (35%)	< 0.001	422 (26.8%)	457 (29.1%)	0.177
DM	628 (9.1%)	386 (11.5%)	< 0.001	139 (8.8%)	151 (9.6%)	0.498
Creatinine, μmol/L	75.7 ± 20.6	83.6 ± 29.5	< 0.001	77.1 ± 17.3	77.8 ± 19.7	0.325
eGFR	93.0 ± 22.1	87.3 ± 25.3	< 0.001	91.5 ± 20.7	91.0 ± 24.1	0.566
BUN	6.1 ± 2.1	7.0 ± 2.8	< 0.001	6.2 ± 1.9	6.3 ± 1.8	0.063
Albumin baseline	40.6 ± 3.3	40.1 ± 3.3	< 0.001	40.0 ± 3.4	39.9 ± 3.5	0.162
Hemoglobin, g/dL	136.5 ± 14.6	137.3 ± 15.9	0.005	134.7 ± 17.0	133.6 ± 18.7	0.093
WBC, 1000/μL	6.4 ± 2.0	6.5 ± 2.2	0.033	6.4 ± 2.4	6.4 ± 2.0	0.532
Elevated RDW, %	0.3 (0.0, 0.6)	0.5 (0.1,1.0)	< 0.001	0.3 (0.0,0.7)	0.5 (0.1,1.1)	< 0.001
Platelet fl	188.8 ± 56.7	177.3 ± 56.4	< 0.001	189.2 ± 62.1	178.2 ± 58.7	< 0.001
BMI, kg/m^2^	22.9 ± 3.0	23.5 ± 3.2	0.013	22.6 ± 3.2	23.3 ± 3.7	< 0.001
SUA	351.0 ± 104.7	394.0 ± 138.6	< 0.001	355.6 ± 101.0	377.0 ± 108.6	< 0.001
NYHA functional class						
I-II	3420 (49.5%)	1370 (40.7)	< 0.001	755 (48.0%)	610 (38.8%)	< 0.001
III-IV	3489 (50.5%)	1995 (59.3)	< 0.001	818 (52.0%)	963 (61.2%)	< 0.001
LVEF%	62.4 ± 8.3	61.4 ± 19.3	0.071	62.1 ± 8.1	61.3 ± 9.4	0.092
CPB time (min)	81.0 (65.0, 105.0)	101.0 (78.0, 128.0)	< 0.001	80.5 (65.0,105.0)	99.0 (78.0,126.0)	< 0.001
ACC time (min)	48.0 (36.0, 67.0)	58.0 (43.0, 78.0)	< 0.001	48.0 (37.0,67.0)	55.0 (41.0,78.0)	< 0.001
Pre-operative coronary angiography n (%)	2543 (36.8)	1528 (45.4)	< 0.001	690 (43.9%)	617 (39.2%)	0.009
Time interval between coronary angiogram and cardiac surgery (days)	4 (2, 6)	4 (2, 6)	0.212	3 (2,6)	3 (2,6)	0.981

*AKI* acute kidney injury, *BMI* body mass index, *DM* diabetes mellitus, *WBC* white blood cell, *RDW* red cell distribution width, *BUN* blood urea nitrogen, *eGFR* estimated glomerular filtration rate *SUA* serum uric acid, *NYHA* New York Heart Association, *LVEF* left ventricular ejection fraction, *CPB* cardiopulmonary bypass, *ACC* Aortic cross-clamp

### Risk factors for the development of CS-AKI

All the variables recorded in Table 1 were put into a univariate logistic regression model and the detailed results are presented in Table 2. The odds ratios of CS-AKI in the unmatched cohort for the independent risk factors that were computed from the multivariate logistic regression model are shown in Table 3. The independent risk factors that were computed from the multivariate logistic regression model were age (OR = 1.036, 95% CI: 1.029–1.042, *P* <  0.001), male (OR = 1.873, 95% CI: 1.622–2.164, *P* <  0.001), BMI (OR = 1.035, 95% CI: 1.016–1.055, *P* <  0.001), elevated RDW (OR = 1.302, 95% CI: 1.209–1.401, *P* <  0.001), BUN (OR = 1.076, 95% CI: 1.041–1.112, *P* <  0.001), SUA (OR = 1.002, 95% CI: 1.001–1.003, *P* <  0.001), CPB time (additional 30 min) (OR = 1.627 95% CI: 1.491–1.775, *P* <  0.001).

**Table 2:** Univariate logistic regression analysis of risk factors for CS-AKI

	Before Propensity Matching			After Propensity Matching		
	OR	95%CI	*P*-value	OR	95%CI	*P*-value
Age	1.030	1.027–1.034	< 0.001	0.999	0.993–1.005	0.782
Male	1.825	1.680–1.994	< 0.001	0.869	0.754–1.002	0.754
BMI	1.066	1.051–1.081	< 0.001	1.063	1.037–1.090	< 0.001
HTN	1.588	1.452–1.736	< 0.001	1.117	0.956–1.305	0.164
DM	1.305	1.145–1.487	< 0.001	1.095	0.860–1.395	0.46
Pre-operative coronary angiography	1.421	1.313–1.553	< 0.001	0.826	0.717–0.952	0.08
Hb	1.004	1.001–1.007	< 0.001	0.997	0.993–1.001	0.093
WBC	1.019	1.002–1.042	< 0.001	0.990	0.958–1.022	0.533
Plt	0.996	0.996–0.997	0.633	0.997	0.996–0.998	0.601
Elevated RDW	1.497	1.419–1.578	< 0.001	1.192	1.126–1.262	< 0.001
BUN	1.178	1.147–1.193	< 0.001	1.036	0998–1.076	0.064
Scr	1.015	1.013–1.017	< 0.001	1.002	0.998–1.006	0.325
eGFR	0.989	0.987–0.991	0.002	0.999	0.996–1.002	0.004
UA	1.003	1.003–1.004	< 0.001	1.002	1.001–1.003	< 0.001
Alb	0.954	0.941–0.965	< 0.001	0.986	0.996–1.006	0.162
CPB time (every 30 min)	1.498	1.439–1.559	< 0.001	1.490	1.385–1.603	< 0.001
ACC time (every 20 min)	1.295	1.248–1.343	< 0.001	1.253	1.176–1.336	< 0.001

*AKI* acute kidney injury, *BMI* body mass index, *HTN* hypertension, *DM* diabetes mellitus, *Hb* hemoglobin, *WBC* white blood cell, *Plt* platelet, *RDW* red cell distribution width, *BUN* blood urea nitrogen, *Scr* serum creatinine, *eGFR* estimated glomerular filtration rate, *SUA* serum uric acid, *CPB* cardiopulmonary bypass, *ACC* Aortic cross-clamp

**Table 3:** Multivariate logistic regression analysis of risk factors for CS-AKI in the unmatched Cohort

	*OR*	*95%* CI	*P-*value
Age	1.036	1.029–1.042	< 0.001
Male	1.873	1.622–2.164	< 0.001
BMI	1.035	1.016–1.055	< 0.001
Elevated RDW	1.302	1.209–1.401	< 0.001
BUN	1.076	1.041–1.112	< 0.001
SUA	1.002	1.001–1.003	< 0.001
CPB time (every 30 min)	1.627	1.491–1.775	< 0.001

*AKI* acute kidney injury, *BMI* body mass index, *RDW* red cell distribution width, *BUN* blood urea nitrogen, *SUA* serum uric acid, *CPB* cardiopulmonary bypass

### The association between elevated RDW and the development and prognosis of CS-AKI

The AKI group had a higher level of elevated RDW than the non-AKI group[0.5% (0.1%, 1.0%) vs 0.3% (0, 0.6%), *P* <  0.001]. Each 0.1% increment in elevated RDW was associated with a 1.1% higher risk of CS-AKI. The elevated RDW were 0.4% (0.1%, 0.9%), 0.5% (0.2%, 1.0%), 0.8% (0.3%, 1.7%) in stages 1, 2 and 3 of AKI, respectively. An increment in elevated RDW was associated with having a higher stage of AKI (*P* = 0.02). The patients were divided into two groups according to whether the RDW baseline was ≥16.0%. The incidence of CS-AKI in the RDW group (> 16.0%) was significantly higher than in the RDW group (≤ 16.0%, 33.9% vs 38.4%, *P* <  0.001). The adjusted odds ratio of CS-AKI development in the RDW group > 16.0% was 1.67-fold, compared with RDW ≤ in the 16.0% group. Among patients with CS-AKI, the level of elevated RDW in non-survivors was higher than survivors[1.1% (0.4%, 2.0%) vs 0.4% (0.1%, 0.9%), *P* <  0.001]. There was no significant difference in elevated RDW between non-survivors and survivors without CS-AKI [0.4% (− 0.1%, 0.9%) vs 0.3% (0.0, 0.6%), *P* = 0.875]. A 0.1% increase in elevated RDW was associated with a 0.24% higher risk of within-hospital mortality in those patients with CS-AKI.

### Bivariate correlation analyses of elevated RDW and various clinical and laboratory parameters of the study population in the unmatched cohort

The results of bivariate correlation analyses showed that there was a positive correlation between elevated RDW and age (*r* = 0.188, *P* <  0.001), HTN (*r* = 0.080, *P* <  0.001), DM (*r* = 0.052, *P* <  0.001), BUN(*r* = 0.039, *P* <  0.001), CPB time (*r* = 0.159, *P* <  0.001), ACC time (*r* = 0.136, *P* <  0.001), AKI stage (*r* = 0.171, *P* <  0.001) and a negative correlation with male (*r* = − 0.096, *P* <  0.001), BMI(*r* = − 0.045, *P* = 0.01), hemoglobin (*r* = − 0.147, *P* <  0.001), Albumin (*r* = − 0.047, *P* <  0.001), and eGFR (*r* = − 0.105, *P* <  0.001) (see Table 4).

**Table 4:** Bivariate correlation analyses of elevated RDW and various clinical and laboratory parameters of the study population in the umatched Cohort

	*r*	*P*-value
Age	0.188	< 0.001
Male	−0.096	< 0.001
BMI	−0.045	0.01
HTN	0.098	< 0.001
DM	0.052	< 0.001
Hb	−0.147	< 0.001
WBC	−0.010	0.292
Alb	−0.047	< 0.001
BUN	0.039	< 0.001
SCr	0.020	0.040
eGFR	−0.105	< 0.001
SUA	−0.010	0.292
CPB time	0.159	< 0.001
ACC time	0.136	< 0.001
AKI stage	0.171	< 0.001

*BMI* body mass index, *HTN* hypertension, *DM* diabetes mellitus, *Hb* hemoglobin, *WBC* white blood cell, *Alb* albumin, *BUN* blood urea nitrogen, *SCr* serum creatinine, *eGFR* estimated glomerular filtration rate, *SUA* serum uric acid, *CPB* cardiopulmonary bypass, *ACC* Aortic cross-clamp, *AKI* acute kidney injury

#### Propensity-matched cohort

Propensity score matching created a matched cohort of 1573 in each group. In this matched cohort, few differences remained in non-AKI and AKI groups (Table 1). The results of univariate logistic regression model are presented in Table 2. The odds ratios of CS-AKI in the matched cohort for the independent risk factors that were computed from the multivariate logistic regression model are shown in Table 5. In the matched cohort, the elevated RDW in AKI patients was higher than in patients without AKI (0.3% (0.0%, 0.7%) vs 0.5% (0.1, 1.1%), *P* <  0.001). The elevated RDW incidences were 0.4% (0.1%, 0.9%), 0.6% (0.2%, 1.1%) and 1.1% (0.3%, 2.1%) in stage 1, 2 and 3 AKI patients (*P* <  0.001). Among patients with CS-AKI, the level of elevated RDW in non-survivors was higher than in survivors [1.2% (0.5%, 2.3%) vs 0.5% (0.1%, 1.0%), *P* <  0.001] and a 0.1% increase in elevated RDW was associated with a 0.24% higher risk of within-hospital mortality in patients with CS-AKI.

**Table 5:** Multivariate logistic regression analysis of risk factors for CS-AKI in the matched Cohort

	*OR*	*95%* CI	*P-*value
BMI	1.078	1.046–1.111	< 0.001
Elevated RDW	1.159	1.074–1.251	< 0.001
eGFR	1.006	1.001–1.010	0.015
SUA	1.002	1.001–1.003	< 0.001
CPB time (every 30 min)	1.493	1.363–1.635	< 0.001

*AKI* acute kidney injury, *BMI* body mass index, *RDW* red cell distribution width, *eGFR* estimated glomerular filtration rate, *SUA* serum uric acid, *CPB* cardiopulmonary bypass

### Receiver-operating characteristic curve analysis for prediction of the development and prognosis of CS-AKI in the matched cohort by elevated red cell distribution width level

To assess discrimination of RDW for all causes of hospital mortality, we used receiver-operating characteristic (ROC) analysis and determined the area under the curve (AUC). The cut-off value of elevated RDW for predicting CS-AKI was 0.30%. The AUC value was 0.605 (95% CI: 0.586–0.625, *P* <  0.001) and the sensitivity, specificity, positive likelihood ratio and negative likelihood ratio of elevated RDW were 51.6%, 63.3%, 1.41 and 0.76, respectively.

Elevated RDW had moderate discriminative power for predicting the death of CS-AKI patients. The cut-off value of elevated RDW for predicting death of CS-AKI patients was 0.75%; AUC value 0.716 (95% CI: 0.640–0.764, *P* <  0.001) and the sensitivity, specificity, positive likelihood ratio and negative likelihood ratio of elevated RDW were 71.4%, 65.5%, 1.53 and 0.83, respectively.

## Discussion

The main finding of the present study was the establishment of an independent association between elevated RDW and in-hospital mortality with CS-AKI. An elevated RDW remains a significant predictor for the severity and mortality of CS-AKI patients following multivariable adjustments. However, there was no clear evidence to show that the elevated RDW was a significant predictor for the development of CS-AKI and there was no significant effect modification between an elevated RDW and in-hospital mortality in those patients without CS-AKI.

A previous study showed that RDW is a strong and independent prognostic predictor of mortality and morbidity in patients with CKD, which described RDW changes in CKD patients undergoing hemodialysis for the first time [12]. Recent research indicates that a higher RDW was independently associated with increased cardiovascular disease (CVD) mortality in peritoneal dialysis (PD) and in end stage renal disease (ESRD) patients [13, 14]. Mario Sičaja et al. found that for each 1% point increase in the RDW level the one-year all cause mortality risk was increased by 54% in patients requiring chronic dialysis [15].

Recent clinical research on RDW and AKI have focused on RDW and contrast-induced AKI in percutaneous coronary interventions (CI-AKI). The results showed that RDW has become a recent target of investigations into new predictors of kidney function and mortality after percutaneous coronary interventions (PCI) [2, 16–18]. The study by Atsushi Mizuno et al. analyzed 102 patients with ST-elevation myocardial infarction and found that RDW was an independent variable predicting CI-AKI and has additional value to the Mehran risk score for predicting contrast-induced (CI)-AKI [19]. Other researchers have investigated the association between RDW and CI-AKI and suggested that RDW was associated independently with the development of CI-AKI and may well be a useful marker in CI-AKI risk stratification [16, 20].

To the best of our knowledge, our study is the first research in the literature that has explored the association between elevated RDW and CS-AKI. However, it needs more research to establish an association between elevated RDW and CS-AKI incidence. The mechanisms underlying the association between higher RDW and poor prognosis have not been clearly determined. Several possible explanations can be considered in cardiac surgery patients. Hemolysis, blood loss, hypothermia, ischemia, and perfusion injury as well as neutrophil activation during CPB play a pivotal role in oxidative stress and the associated activation of inflammation. In particular, ischemia and reperfusion injury during cardiac surgery can lead to the generation of pro-inflammatory mediators [21–24]. In our research, the results of bivariate correlation analyses showed that there was a positive correlation between elevated RDW and the CPB and ACC times. Inflammation may play a role in the regulation of RDW by direct myelo-suppression of erythroid precursors, decreasing erythropoietin production, reducing the bioavailability of iron and by promoting erythropoietin resistance and red cell apoptosis [25–29].

Related research [30–34] has demonstrated that oxidative stress increases anisocytosis by disrupting erythropoiesis and altering blood cell membrane deformability and thus the red blood cell circulation lifetime, ultimately leading to an increase in RDW. In addition, during CPB, ischemia and reperfusion play a pivotal role in oxidative stress by initiating a series of biochemical events [22]. However, in our study, we were not able to adjust the analysis to include inflammatory markers, C-reactive protein (CRP) and other factors, as these were not routinely measured at patient admission.

The renin-angiotensin-aldosterone system (RAAS) is activated by arterial underfilling caused by CPB, heart failure, shock and so on after cardiac surgery. The activation of the RAAS system and adrenergic hormone release could cause increased RDW with erythropoiesis, resulting in a poor prognosis [34]. There is evidence suggesting that angiotensin II acts as a growth factor for erythroid precursors as well as an erythropoietin secretagogue resulting in an increment in RDW due to macrocytosis from skipped cell divisions [35].

RDW is increased in conditions such as malnutrition and deficiencies such as in vitamin B12 [36], iron [37] or folic acid [38], increased red cell destruction (such as hemolysis), or after blood transfusion. Previous research has reported that RDW was correlated negatively with nutrition in heart failure [39] and HD patients [13]. This latter study showed that elevated RDW was negatively correlated with the albumin level. Patients with malnutrition have a higher risk of infection and adverse outcomes and elevated RDW and poor prognosis may relate to the fact that there is an interaction between malnutrition and inflammation.

The present research work has several limitations. First, it was a retrospective design from a single central pool of patients. Second, we did not measure hematopoietic factors (including iron, vitamin B12, folate, etc.) that may influence RDW. Third, though neurohumoral activation and inflammation may be a mechanistic link between elevated RDW and poor outcomes, we did not assess laboratory markers including CRP, other pro-inflammatory cytokines, norepinephrine and angiotensin II. Despite these limitations, a major strength of the present study is that it had a sufficient number of patients undergoing cardiac surgery to ensure adequate reliability of our incidence and mortality estimates. Future studies of a larger cohort of single center pool patients will be required to confirm the results of the present investigation.

## Conclusion

The results of the present study suggest, that an elevated RDW might be an independent prognostic factor for the severity and poor prognosis of CS-AKI.

## Abbreviations

ACC: Aortic cross-clamp
AKI: Acute kidney injury
AUC: Area under the curve
BMI: Body mass index
BUN: Blood urea nitrogen
CI-AKI: AKI in percutaneous coronary interventions
COPD: Chronic obstructive pulmonary disease
CPB: Cardiopulmonary bypass
CRP: C-reactive protein
CS-AKI: AKI in patients undergoing cardiac surgery
CVD: Cardiovascular disease
DM: Diabetes mellitus
eGFR: Estimated glomerular filtration rate
ESRD: End stage renal disease
IQR: Interquartile range
KDIGO: Kidney Disease Improving Global Outcomes
LOS: Length-of-stay
MCV: Mean corpuscular volume
ORs: Odds ratios
PCI: Percutaneous coronary interventions
PD: Peritoneal dialysis
RAAS: Renin-angiotensin-aldosterone system
RDW: Red cell distribution width
ROC: Receiver-operating characteristic
RRT: Renal replacement therapy
Scr: Serum creatinine
SUA: Serum uric acid
WBC: White blood cells
